# Colorectal Malignancy in a Prospective Irish Inflammatory Bowel Disease Population 15 Years Since Diagnosis: Comparison with the EC-IBD Cohort

**DOI:** 10.1155/2017/4946068

**Published:** 2017-09-24

**Authors:** Mary Shuhaibar, Colm O'Morain

**Affiliations:** ^1^Department of Gastroenterology/General Medicine, Adelaide and Meath Hospital Incorporating the National Children Hospital, Tallaght, Ireland; ^2^Department of Clinical Medicine, Trinity College Dublin, Dublin 2, Ireland

## Abstract

**Background and Aim:**

As part of the EC-IBD prospective inception cohort study, we had unique opportunity to follow up our patients since diagnosis in the early 1990s.

**Patients and Methods:**

All patients from the greater Dublin area (*n* = 192) were followed up from inception between 1991 and 1993 until the 30 September 2009. Patients who developed malignancies were logged electronically with verification of the site and histology.

**Results:**

Of the initial 192 patients, 133 were included in the 15-year follow-up. Of those, 80 (60.2%) had UC and 53 (39.8%) had CD. There were 82 (61.7%) males and 51 (38.3%) females. Six patients had extraintestinal malignancy; however, there was no CRC related to IBD noted in our cohort. Four of the 6 identified cases had UC (64%) with a mean age of 54.25 years at the time of cancer diagnosis, whereas the two CD patients had a mean age of 51.5 years at the time of cancer diagnosis.

**Conclusion:**

CRC was not observed in our cohort. The six extraintestinal malignancies did not show significant relation to IBD. The high total colectomy rate (in the prebiological therapy era) may have contributed to low malignancy rate.

## 1. Introduction

Inflammatory bowel disease with its two main forms ulcerative colitis (UC) and Crohn's disease (CD) has been associated with variable risk of malignancy. The first report of intestinal cancer in IBD patients was published over 80 years ago [1]. Since then, there have been multiple studies attempting to identify the true risk of malignancy in IBD patients. The observed risk was noted to be higher in referral centres [2–6] compared to population-based studies [7–10], and yet in some population-based studies [11–14], the overall risk of colorectal cancer (CRC) was comparable to the background population.

Part of the discrepancy may have resulted from the variation in population constellations, diagnostic ascertainment, and treatment strategies at the different specialised centres compared to population-based cohorts. Although the risk of CRC in UC patients is thought to increase with disease duration and extent, this may not have been the case in some centres where early colectomy was provided for patients with severe diseases in an attempt to control their symptoms early on in the disease course, especially in the prebiological therapy era.

The overall reported cumulative incidence of CRC in IBD patients from different studies ranged between 0.6% and 17% [6, 8, 15–20].

A recent meta-analysis of over 100 publications calculated CRC risk in patients with left-sided UC or pancolitis as 2% by 10 years, 8% by 20 years, and 18% by 30 years of diagnosis. Higher risk was reported in those with pancolitis or severe inflammation [21, 22]. However, those with proctitis and proctosigmoiditis are probably not at increased risk of CRC compared to the general population [23]. CD-associated CRC is observed to have a similar timeline to UC-associated CRC [5, 24]. The median duration of the disease before the CRC diagnosis was comparable for CD and UC at 15 and 18 years, respectively. The median age at CRC diagnosis was 55 years in CD compared to 43 years in UC patients. Risk factors for the development of CRC include familial occurrence of sporadic CRC, comorbidity with primary sclerosing cholangitis [25], and young age of colitis onset.

Furthermore, there appears to be an increased risk of malignancy outside the intestinal tract in IBD patients that can be as a consequence of the disease itself or as a result of the immune-modulatory therapy used. There have been reports of leukaemia in UC patients as well as leukaemia and lymphoma in CD patients in different cohorts [10, 26, 27].

The aim of this study was firstly to determine the occurrence and characteristics of IBD-related colorectal dysplasia and cancer of the gut and the frequency of extraintestinal malignancy in the first 15 years after IBD diagnosis in a prospective Irish inception cohort followed up since 1991.

Secondly, we wanted to compare our findings to those of other northern and southern European centres that made part of the European Collaborative IBD Study Group that was established in 1991–1993 and participated in this follow-up study.

## 2. Materials and Methods

### 2.1. Patients and Centres

As a major contributor to the European Collaborative Study Group of Inflammatory Bowel Disease (EC-IBD), Ireland recruited 192 patients (from the greater Dublin area) as part of a prospective inception, population-based cohort of uniformly diagnosed 2201 IBD patients within 20 well-described European geographical areas. Study areas and participating populations were described in detail previously [28, 29]. Our results were incorporated as one of the nine out of the original 20 EC-IBD centres that contributed for this 15-year follow-up study from inception until 30 September 2009. The Irish cohort was in a position to contribute to this study as a result of this research follow-up work, despite being not able to previously contribute to the 10-year data analysis for logistical reasons. There were other centres with logistical issues limiting their contribution at this time. Of note, since each of the participating centres individually met the initial inclusion criteria, there was no negative effect on the population-based cohort from those centres that did not participate in this follow-up.

The aim was to investigate the rate of CRC and dysplasia in this IBD cohort. Patients were excluded from this study if they had been lost to follow-up or had colectomy for reasons other than colorectal dysplasia or cancer.

All participating centres applied for local ethical permissions, and approval had been granted. There were two electronic internet-based facilities available for all participating centres for the input of data by all scientific working groups.

### 2.2. Data Collection

An electronic physician per patient follow-up form was completed per patient follow-up and linked up to the coordinating centre. Patients' records were reviewed. The diagnosis of dysplasia was established on routine biopsies, as in clinical practice, but not in a central pathology laboratory. However, in each participating centre, two expert gastropathologists confirmed dysplasia in the original specimen biopsy.

### 2.3. Definitions

Neoplasia expected rates were calculated using aggregate codes for each diagnosis as listed in both the ICD-9 and ICD-10 of the WHO Mortality Databases from 1995 to 1998 [30]. The expected numbers were calculated assuming that EC-IBD patients have the same risk of cancer diagnosis as the general population in their respective countries. Information for cancer rates from the IARC (International Association of Registries of Cancer) was used for comparisons [31]. However, there was no such a database available for dysplasia.

### 2.4. Statistical Analysis

The collected information from questionnaires was analysed centrally at the University of Maastricht, the Netherlands. The chi-square test and *t*-test were performed to identify possible differences in all demographic and clinical variables among patients with and without cancers and colorectal dysplasia.

The IACR database was used for cancer data across Europe. Statistical analysis for relative cancer risk (95% confidence intervals—CI) was made using assumptions for calculations of the estimated and observed IBD intestinal and extraintestinal cancers in the different EC-IBD centres. In the null hypothesis, patients of this multicentre cohort had the same risk for cancer as the matched general population in their respective countries.

The SPSS 12.0 statistical software package was used for the analysis.

## 3. Results

There were 192 Irish patients included at the initial cohort between 1991 and 1993, and available data on 133 patients of the 192 Irish patients were included in the 15-year follow-up study. Of the 133 patients, 80 (60.2%) had UC and 53 (39.8%) had CD, which accounted for 45.2% and 50% of the contributing northern UC and CD overall cohort, respectively. There were 82 (61.7%) males and 51 (38.3%) females. 71% of the Irish IBD cohort were diagnosed under 40 years of age, and in particular, 62% of CD patients were diagnosed under 30 years of age compared to 33% of the UC group for the same age.

Six patients had extraintestinal malignancy; however, there was no CRC related to IBD noted in our cohort (Table 1). Four of the six cases had UC (64%) with a mean age of 54.25 years at the time of cancer diagnosis, although the mean age of the two CD patients was 51.5 years at the time of cancer diagnosis.

On the one hand, in the UC group, 53% had distal colitis (proctitis and proctosigmoiditis), 30% had inflammation of the left colon (up to splenic flexure), and 17% were diagnosed with pancolitis—at the time of this follow-up study. On the other hand, in the CD group, 33% had colonic disease distribution, 21% had ileo-colonic inflammation, and 46% had a disease which was confined to the terminal ileum.

There were nine centres in total providing data for the EC-IBD follow-up study with 681 IBD patients analysed (445 UC and 236 CD) [32]. These were divided into southern European and northern European countries represented by Heraklion (Greece), Ioannina (Greece), Vigo (Spain), Beer Sheva (Israel), Cremona (Italy), and Reggio Emilia (Italy) for the south and Dublin (Ireland), South Limburg (Netherlands), and Copenhagen (Denmark) for the north (Table 2).

Overall, in the multicentre study, there were 62 patients with 66 cancers diagnosed over the same follow-up period. Two-thirds of these cases were reported in UC patients. Of note, there were no CRCs reported early in disease courses. Extraintestinal cancer was diagnosed in 53 patients. Lung, skin, lymphoma, and breast cancers were the leading four. Although the frequency of extraintestinal cancers appeared higher in southern EC-IBD centres compared to northern ones, the difference failed to reach statistical significance. The frequency of observed versus expected cancers was calculated, and comparison between our centre to the mean of the northern and southern centres was made (Tables 3 and 4).

In the Irish cohort, there was no evidence of dysplasia in those who underwent repeat endoscopy during the follow-up period based on their reviewed histology reports.

Compared with the other centres, the Irish cohort has a much lower relative risk of developing either CRC or non-CRC malignancy in the studied population. However, in the multicentre population, there were 17 patients who were found to have colorectal dysplasia varying between low-grade dysplasia in 12 patients and high-grade dysplasia in the remaining five patients. Different surgical management was applied to the high-grade dysplasia group depending on severity and multidisciplinary team opinion. Only one colonic resection showed evidence of adenocarcinoma.

## 4. Discussion

The follow-up study on the EC-IBD cohort showed an overall prevalence of intestinal and extraintestinal cancers of 9.1%, while the prevalence of CRC and colorectal dysplasia was 1.3% and 2.5%, respectively [32]. A recent study of 600 UC patients with a 30-year surveillance program showed a cumulative CRC and colonic dysplasia prevalence of 6.3% and 11%, respectively [33].

Another follow-up study for both UC and CD patients from Olmsted County, Minnesota, showed an overall intestinal cancer prevalence of 2.1%, with increased risk in severe UC patients and 40-fold higher risk of small bowel cancers in CD patients [34].

The strength of the present study is that it is a population based, multicentre, prospective cohort with uniform diagnostic criteria for inclusion. All patients were followed up simultaneously apart from those lost due to death, lost to follow-up, or requiring colectomy for reasons other than dysplasia or malignancy.

On the other hand, there were some weaknesses related to variable number of patients during the follow-up from the different centres, and not all original centres were in a position to contribute to this 15-year study period, as a completeness rate of at least 50% was asked of those who participated. Furthermore, treatment options including surgery and maintenance aminosalicylate therapy as a possible chemopreventative agent for CRC, and interval CRC screening in IBD patients may vary across Europe. Collaborative guidelines were put forward to recommend surveillance colonoscopy frequencies in IBD patients [35].

Although the EC-IBD study observed 66% of the extra intestinal cancers in UC patients, the data did not support any statistical increase in risk of developing those cancers in general UC patients. Multiple studies noted, similarly, that although severe UC may increase the risk of CRC, it did not seem to be associated with an increased risk of cancer in general [7, 27, 36]. Furthermore, UC patients were more likely to develop IBD-related CRC or dysplasia than their CD counterparts [37, 38]. In our cohort, only half of cancer patients were on maintenance treatment with topical aminosalicylate and corticosteroid therapy despite some reports of their role in reducing CRC risk in UC patients [39].

Of note, at our centre in Dublin, there was no intestinal malignancy found similar to centres in Greece, Portugal, and Italy, but yet, extraintestinal cancers were prevalent. This in part may relate to the disease phenotype, in addition to the rate of surgery. Nearly 14% of UC patients had colectomy in the first five years of diagnosis.

There were very few patients taking immune modulators in the form of azathiopurine with its controversial side effects [40]. If we compare the overall results from northern and southern EC-IBD centres, we notice that northern centres have a higher tendency for CRC while the southern ones have a tendency for extraintestinal cancers, although this is not statistically significant. Whether this indicates different disease phenotypes or just reflects the disproportionate sample size between those centres remains to be seen.

We note from the findings that there were 9.7% with blood-related malignancy as primary or coexisting diagnosis, which can be of doubtful significance, especially in light of conflicting reports. While some case series identified an increased risk of lymphoma and or leukaemia in UC patients [26, 41], other population-based studies from Scandinavia and Canada did not show any increased risk [7, 10, 14, 36, 42].

In conclusion, this study showed that no CRC was observed in this IBD cohort over a period of 15 years of follow-up, although when compared to the multicentre EC-IBD, there was CRC and related dysplasia prevalence of 1.3% and 2.5%, respectively. This may have resulted from rather high surgical rates at the initial decade of IBD diagnosis, particularly colectomy rates for severe UC patients in the prebiologic era.

## Figures and Tables

**Table 1:** Intestinal and extraintestinal cancer types in Ireland and ICD-10 coding.

Diagnosis	Sex	Year of birth	Year of IBD diagnosis	Year of cancer diagnosis	Cancer type	ICD-10 coding
UC	M	1945	1992	2003	Non-Hodgkin lymphoma	37
UC	M	1958	1992	2007	Phaeochromocytoma	33
UC	F	1932	1992	2002	Melanoma	22
UC	F	1960	1992	2000	Lung cancer	19
CD	F	1962	1992	2003	BCC (basal cell carcinoma)	22
CD	M	1943	1992	2005	Prostate cancer	29

UC: ulcerative colitis; CD: Crohn's disease; F: female; M: male.

**Table 2:** Summary of EC-IBD intestinal and extraintestinal cancer.

Centre	Number of patients	Number of patients	Ulcerative colitis	Crohn's disease	Males	Females	Intestinal cancers	Intestinal cancers	Extraintestinal cancers^∗^	Extraintestinal cancers
	1991–1993	1991–2009	2009	2009	2009	2009	1993–2004	1993–2009	1993–2004	1993–2009
Copenhagen	153	124	82	42	68	56	2	3 (2 F/1 M)	6	16 (7 F/9 M)
South Limburg	222	159	95	64	74	85	2	2 (1 F/1 M)	4	4 (3 F/1 M)
Total north (excl Dublin)	375	283	177	106	142	141	4	5 (3 F/2 M)	10	20 (10 F/10 M)

Dublin (% of total northern contributing centres)	192 (51%)	133 (47%)	80 (45.2%)	53 (50%)	82 (57.7%)	51 (36%)	N/A	0	N/A	6 (3 F/3 M) (30%)

Vigo	100	63	36	27	38	25	0	0	4	12 (2 F/10 M)
Beer Sheva	64	18	12	6	11	7	0	1 (M)	6	7 (5 F/2 M)
Ioannina	43	36	32	4	20	16	0	0	0	0
Cremona	53	26	18	8	14	12	0	0	1	3 (3 M)
Heraklion	74	53	45	8	31	22	0	0	3	3 (1 F/2 M)
Reggio Emilia	86	69	45	24	36	33	1	3 (1 F/2 M)	1	2 (1 F/1 M)
All south	420	265	188	77	150	115	1	4 (1 F/3 M)	15	27 (9 F/18 M)
	1365	681	445	236	374	307	8	9 (4 F/5 M)	30	53 (22 F/31 M)

Total (% follow-up)	−100	−50								

**Table 3:** All cancer types.

Centre	Total patients (2009)	Observed^∗^	Expected^∗∗^	Relative risk	Lower CI	Upper CI
Dublin	133	6	0.29	0.68	0.31	1.51
All north mean	316	5	0.90	1.72	0.72	4.10
All south mean	265	4	1.54	1.50	0.57	3.98

^∗^In cases with more than one cancer, colorectal cancer was regarded as the principal cancer diagnosis and cancers of unknown primary origin were classified as extraintestinal. ^∗∗^Assumptions for these calculations were as follows: (1) the expected numbers were calculated assuming that the patients of the EC-IBD cohort have the same risk of cancer diagnosis as the general population in their respective country; (2) the EC-IBD cohort has been assembled in the period 1991–1993, and expected numbers have been collectively calculated from the contributing centres for the 15-year follow-up period; and (3) cancer incidence rates for each study centre were obtained from the ΙΑCR databases (with actual and estimated cancer incidence rates for European countries in 2008). CI = confidence interval.

**Table 4:** CRC and extraintestinal cancers in the EC-IBD study mean related to the Irish cohort.

Cancer	Total patients (2009)	Observed	Expected	Relative risk (RR)	Confidence interval (CI) (low-high)
*CRC*					
Dublin	133	0	0.01	0	
All north	316	5	0.01	1.72	0.72–4.1
All south	265	4	0.01	1.5	0.57–3.98
*Non-CRC*					
Dublin	133	6	0.06	0.78	0.35–1.72
All north	316	26	0.06	0.88	0.88–1.85
All south	265	27	0.06	1.7	1.19–2.43

CRC: colorectal cancer.

## References

[1] Crohn B. B., Rosenberg H. (1925). The sigmoidoscopic picture of chronic ulcerative colitis (non specific). *The American Journal of the Medical Sciences*.

[2] Weedon D. D., Shorter R. G., Ilstrup D. M., Huizenga K. A., Taylor W. F. (1973). Crohn’s disease and cancer. *The New England Journal of Medicine*.

[3] Gyde S. N., Prior P., Macartney J. C., Thompson H., Waterhouse J. A., Allan R. N. (1980). Malignancy in Crohn’s disease. *Gut*.

[4] Gillen C. D., Andrews H. A., Prior P., Allan R. N. (1994). Crohn’s disease and colorectal cancer. *Gut*.

[5] Gillen C. D., Walmsley R. S., Prior P., Andrews H. A., Allan R. N. (1994). Ulcerative colitis and Crohn’s disease: a comparison of the colorectal cancer risk in extensive colitis. *Gut*.

[6] Greenstein A. J., Sachar D. B., Smith H., Janowitz H. D., Aufses A. H. (1981). A comparison of cancer risk in Crohn’s disease and ulcerative colitis. *Cancer*.

[7] Bernstein C. N., Blanchard J. F., Kliewer E., Wajda A. (2001). Cancer risk in patients with inflammatory bowel disease: a population-based study. *Cancer*.

[8] Ekbom A., Helmick C., Zack M., Adami H. O. (1990). Ulcerative colitis and colorectal cancer: a population-based study. *The New England Journal of Medicine*.

[9] Gilat T., Fireman Z., Grossman A. (1988). Colorectal cancer in patients with ulcerative colitis: a population study in central Israel. *Gastroenterology*.

[10] Karlén P., Löfberg R., Broström O., Leijonmarck C. E., Hellers G., Persson P. G. (1999). Increased risk of cancer in ulcerative colitis: a population-based cohort study. *The American Journal of Gastroenterology*.

[11] Fireman Z., Grossman A., Lilos P. (1989). Intestinal cancer in patients with Crohn’s disease: a population study in central Israel. *Scandinavian Journal of Gastroenterology*.

[12] Jess T., Winther K. V., Munkholm P., Langholz E., Binder V. (2004). Intestinal and extra-intestinal cancer in Crohn’s disease: follow-up of a population-based cohort in Copenhagen County, Denmark. *Alimentary Pharmacology & Therapeutics*.

[13] Persson P. G., Karlen P., Bernell O. (1994). Crohn’s disease and cancer: a population-based cohort study. *Gastroenterology*.

[14] Winther K. V., Jess T., Langholz E., Munkholm P., Binder V. (2004). Long-term risk of cancer in ulcerative colitis: a population-based cohort study from Copenhagen County. *Clinical Gastroenterology and Hepatology: the official clinical practice journal of the American Gastroenterological Association*.

[15] Bansal P., Sonnenberg A. (1996). Risk factors of colorectal cancer in inflammatory bowel disease. *The American Journal of Gastroenterology*.

[16] Ekbom A. (1998). Risk factors and distinguishing features of cancer in IBD. *Inflammatory Bowel Diseases*.

[17] Ekbom A., Helmick C., Zack M., Adami H. O. (1990). Increased risk of large-bowel cancer in Crohn’s disease with colonic involvement. *Lancet*.

[18] Katsanos K. H., Stamou P., Tatsioni A. (2011). Prevalence of inflammatory bowel disease related dysplasia and cancer in 1500 colonoscopies from a referral center in northwestern Greece. *Journal of Crohn's & Colitis*.

[19] Munkholm P., Langholz E., Davidsen M., Binder V. (1993). Intestinal cancer risk and mortality in patients with Crohn’s disease. *Gastroenterology*.

[20] Brostrom O., Lofberg R., Nordenvall B., Ost A., Hellers G. (1987). The risk of colorectal cancer in ulcerative colitis: an epidemiologic study. *Scandinavian Journal of Gastroenterology*.

[21] Eaden J. A., Abrams K. R., Mayberry J. F. (2001). The risk of colorectal cancer in ulcerative colitis: a meta-analysis. *Gut*.

[22] Lennard-Jones J. E., Melville D. M., Morson B. C., Ritchie J. K., Williams C. B. (1990). Precancer and cancer in extensive ulcerative colitis: findings among 401 patients over 22 years. *Gut*.

[23] Levin B. (1992). Inflammatory bowel disease and colon cancer. *Cancer*.

[24] Choi P. M., Zelig M. P. (1994). Similarity of colorectal cancer in Crohn’s disease and ulcerative colitis: implications for carcinogenesis and prevention. *Gut*.

[25] Eaden J. A., Mayberry J. F. (2000). Colorectal cancer complicating ulcerative colitis: a review. *The American Journal of Gastroenterology*.

[26] Greenstein A. J., Gennuso R., Sachar D. B. (1985). Extraintestinal cancers in inflammatory bowel disease. *Cancer*.

[27] Palli D., Trallori G., Bagnoli S. (2000). Hodgkin’s disease risk is increased in patients with ulcerative colitis. *Gastroenterology*.

[28] Wolters F. L., Russel M. G., Sijbrandij J. (2006). Crohn’s disease: increased mortality 10 years after diagnosis in a Europe-wide population based cohort. *Gut*.

[29] Wolters F. L., van Zeijl G., Sijbrandij J. (2005). Internet-based data inclusion in a population-based European collaborative follow-up study of inflammatory bowel disease patients: description of methods used and analysis of factors influencing response rates. *World Journal of Gastroenterology*.

[30] WHO (1992). *The Tenth Revision of the International Statistical Classification of Diseases and Related Health Problems (ICD-10)*.

[31] Breslow M. D., Day N. E. (1986). Statistical Methods in Cancer Research. *The Design and Analysis of Cohort Studies*.

[32] Katsanos K. H., Tatsioni A., Pedersen N. (2011). Cancer in inflammatory bowel disease 15years after diagnosis in a population-based European Collaborative follow-up study. *Journal of Crohn's and Colitis*.

[33] Rutter M. D., Saunders B. P., Wilkinson K. H. (2006). Thirty-year analysis of a colonoscopic surveillance program for neoplasia in ulcerative colitis. *Gastroenterology*.

[34] Jess T., Loftus E. V., Velayos F. S. (2006). Risk of intestinal cancer in inflammatory bowel disease: population-based study from Olmsted County, Minnesota. *Gastroenterology*.

[35] Cairns S. R., Scholefield J. H., Steele R. J. (2010). Guidelines for colorectal cancer screening and surveillance in moderate and high risk groups (update from 2002). *Gut*.

[36] Ekbom A., Helmick C., Zack M., Adami H.-O. (1991). Extracolonic malignancies in inflammatory bowel disease. *Cancer*.

[37] Leidenius M. H., Farkkila M. A., Karkkainen P., Taskinen E. I., Kellokumpu I. H., Hockerstedt K. A. (1997). Colorectal dysplasia and carcinoma in patients with ulcerative colitis and primary sclerosing cholangitis. *Scandinavian Journal of Gastroenterology*.

[38] Marchesa P., Lashner B. A., Lavery I. C. (1997). The risk of cancer and dysplasia among ulcerative colitis patients with primary sclerosing cholangitis. *The American Journal of Gastroenterology*.

[39] Moody G. A., Jayanthi V., Probert C. S., Mac Kay H., Mayberry J. F. (1996). Long-term therapy with sulphasalazine protects against colorectal cancer in ulcerative colitis: a retrospective study of colorectal cancer risk and compliance with treatment in Leicestershire. *European Journal of Gastroenterology & Hepatology*.

[40] Connell W. R., Kamm M. A., Ritchie J. K., Lennard-Jones J. E., Dickson M., Balkwill A. M. (1994). Long-term neoplasia risk after azathioprine treatment in inflammatory bowel disease. *The Lancet*.

[41] Mir-Madjlessi S. H., Farmer R. G., Easley K. A., Beck G. J. (1986). Colorectal and extra-colonic malignancy in ulcerative colitis. *Cancer*.

[42] Mellemkjaer L., Olsen J. H., Frisch M., Johansen C., Gridley G., McLaughlin J. K. (1995). Cancer in patients with ulcerative colitis. *International Journal of Cancer*.

